# The role of plant hormones on the reproductive success of red and brown algae

**DOI:** 10.3389/fpls.2022.1019334

**Published:** 2022-10-19

**Authors:** Toshiki Uji, Hiroyuki Mizuta

**Affiliations:** Laboratory of Aquaculture Genetics and Genomics, Division of Marine Life Science, Faculty of Fisheries Sciences, Hokkaido University, Hakodate, Japan

**Keywords:** 1-aminocylopropane-1-carboxylic acid, abscisic acid, auxin, plant hormone, reactive oxygen species, reproduction, stress tolerance, macroalgae

## Abstract

Seaweeds or macroalgae are important primary producers that serve as a habitat for functioning ecosystems. A sustainable production of macroalgae has been maintained by a diverse range of life cycles. Reproduction is the most dynamic change to occur during its life cycle, and it is a key developmental event to ensure the species’ survival. There is gradually accumulating evidence that plant hormones, such as abscisic acid and auxin, have a role on the sporogenesis of brown alga (*Saccharina japonica*). Recent studies reported that 1-aminocylopropane-1-carboxylic acid, an ethylene precursor, regulates sexual reproduction in red alga (*Neopyropia yezoensis*) independently from ethylene. In addition, these macroalgae have an enhanced tolerance against abiotic and biotic stresses during reproduction to protect their gametes and spores. Herein, we reviewed the current understanding on the regulatory mechanisms of red and brown algae on their transition from vegetative to reproductive phase.

## Introduction

Seaweeds or macroalgae are macroscopic aquatic organisms that independently evolved three lineages that are generally named green, red, and brown algae. Macroalgae produce natural ecosystems and support high levels of biodiversity in coastal zones by providing food, shelter, and habitat for various marine organisms, including invertebrates, fish, mammals, and seabirds (Graham, 2004; Teagle et al., 2017). They are also well-known sources of hydrocolloids, cosmetic compounds, bioremediation agents, and potential biofuels as well as foods for human nutrition (Rajapakse and Kim, 2011; Deepika et al., 2022). In particular, red and brown algae are valuable sources of carbohydrate hydrocolloids (e.g., carrageenan, agar, and alginate), which are used as thickening and gelling agents in food (Rhein-Knudsen and Meyer, 2021).

Many species of macroalgae have heteromorphic (two morphologically distinct generations) or isomorphic (two morphologically similar generations) life cycles with sporophyte and gametophyte phases. Different phases of the same species have abilities to exploit different ecological niches by adapting to environmental factors, such as temperature, light levels, desiccation, or herbivore abundance (Lubchenco and Cubit, 1980; Zupan and West, 1990; Klinger, 1993; Thornber, 2006). Thus, an optimal timing of gametogenesis and sporogenesis is critical for their reproductive success. Consequently, macroalgae are expected to constantly monitor environmental and endogenous signals to control their growth and reproduction. Previous field surveys and laboratory studies suggest that the transition from vegetative to reproductive development is tightly regulated by external environmental factors, such as day length, temperature, and nutrients (Brawley and Johnson, 1992; Agrawal, 2012; Liu et al., 2017). In contrast to external factors, our knowledge on endogenous signaling molecules mediating the transition to reproductive phase remains limited. However, recent studies suggest that plant growth regulators, also known as plant hormones, seem to be important regulatory molecules that control the transition from vegetative to reproductive stage, especially in red and brown algae.

Reproductive tissues occur by degrading cell walls for the release of spores or gametes; thus, they are expected to be vulnerable to abiotic and biotic stresses, such as UV radiation, inadequate temperatures, nutrient limitation, desiccation, pathogen, and herbivore attack. Thus, protection against stress-induced oxidative damage during reproduction is necessary for a successful production of offspring. Recent studies showed that macroalgae enhance their stress tolerance during reproduction to protect their gametes and spores. A deeper understanding on the mechanisms of trade-off that regulate growth, reproduction, and defense responses is important to sustainably produce seaweeds under climate changes and provide useful knowledge for new breeding that could increase the yield in the future. Herein, we reviewed the recent studies on the roles of plant hormones and stress tolerance in the reproduction of red and brown algae.

## Role of plant hormones and stress tolerance in the reproduction of brown algae

Kelps (order Laminariales, class Phaeophyceae) provide structural habitats for numerous ecologically and economically important marine species and provide food, alginates, iodine, and pharmaceutical products (Bartsch et al., 2008). Laminariales typically exhibit a heteromorphic life cycle that alternates between microscopic gametophytes and macroscopic sporophytes (Schiel and Foster, 2006). Mature diploid sporophytes release haploid spores that settle and develop into either female or male gametophytes, which produce eggs or sperms, respectively, to generate diploid sporophytes (Bartsch et al., 2008).

The gametophytes of Laminariales are involved in sex pheromones released from female gametes or eggs (Maier et al., 2001). In contrast, there is evidence that two plant hormones, abscisic acid (ABA) and auxin, are involved in the reproduction of their sporophytes, such as in *Saccharina japonica*, which is an essential ingredient for making dashi (soup stock) as “kombu” in Japanese cuisine. An exogenous application of ABA to sporophyte discs of *S. japonica* promoted the formation of sori (Nimura and Mizuta, 2002), which are reproductive organs with zoosporangia and paraphysis originating from epidermal cells. During reproduction, the sori accumulated endogenous ABA to about five times higher than that in vegetative parts (Nimura and Mizuta, 2002). On the other hand, ABA application inhibited their growth (Nimura and Mizuta, 2002). In addition, a negative correlation between endogenous ABA levels and growth rates of *Laminaria hyperborea* sporophytes was reported, suggesting that ABA acts as a vegetative growth inhibitor in Laminariales (Schaffelke, 1995a; Schaffelke, 1995b). These findings suggest that ABA synthesis triggers the switch to reproductive phases in Laminariales sporophytes.

In contrast, sorus formation was markedly suppressed in the discs of sporophytes of *S. japonica* and *Undaria pinnatifida* in the presence of indole-3-acetic acid (IAA) (Kai et al., 2006), which is the most studied natural auxin in land plants. Consistent with the results, free and conjugated auxin contents were lower in sorus parts than in vegetative parts (Kai et al., 2006). Additionally, red light irradiation inhibited sorus formation with a concomitant decrease in IAA oxidase activity (Mizuta et al., 2007). Previous studies suggest that inhibitors of sporangium formation move from intercalary meristem at the basal to distal frond portions, leading to sorus formation inhibition (Buchholz and Lüning, 1999; Lüning et al., 2000). The sporulation-inhibiting factor might be IAA; however, auxin transports through plant tissues, moving from cell to cell in higher plants, but it remains unknown in brown algae. Although the role of auxins in brown algae is unclear, further investigation on functional interactions between ABA and auxins in Laminariales could provide important findings on the mechanisms underlying the transition of brown algae from vegetative to reproductive phase.

In *S. japonica*, sorus accumulates high levels of phenolics and silicon compared to its vegetative parts (Mizuta and Yasui, 2010; Mizuta and Yasui, 2012). In brown algae, phenolics, which are mainly represented by phlorotannins, have high antioxidant properties and play a defensive role in protecting against herbivory and UV radiation (Lemesheva and Tarakhovskaya, 2018). Similarly, electron microscopic observations revealed that many physodes, which accumulate a large amount of phenolic substances (Schoenwaelder, 2002), appear in paraphyses and zoosporangia of *Saccharina angustata* (Motomura, 1993). In contrast to the role of phlorotannins, the effects of silicon in macroalgae are not well-understood, although silicon in higher plants has been shown to be involved in amorphous silica barrier formation, which can help alleviate both biotic and abiotic stresses (Liang et al., 2007). Silicon is highly deposited in sori, particularly in paraphyses and mucilage caps, which are thought to provide a protective covering for zoosporangia (Fritsch, 1952). Silicate uptake in *S. japonica* sporophytes is activated by iodoperoxidase (IPO) whose activity is promoted in sporophytes treated with ABA (Shimizu et al., 2018; Mizuta et al., 2021). Therefore, phenolics, silicon, and IPO could act as a defensive capacity for *S. japonica* reproduction.

Halogens also play an important role in protecting brown algae from grazing and fungal and bacterial attacks (Al-Adilah et al., 2022). Treatment with oligosaccharides derived from alginate, the main brown algal cell wall polysaccharide, shows that Laminariales has a high capacity to release iodide during oxidative bursts (Shimizu et al., 2018) that induce subsequent defense responses (Küpper et al., 2002; Thomas et al., 2014). In contrast to other antioxidants, the iodine content of *S. japonica* sorus is lower level than that in adjacent vegetative parts, suggesting that the iodide released from sorus contributes to chemical defense in reproductive tissues against fungal and bacterial infection.

## Role of plant hormones and stress tolerance in the reproduction of red algae

A large group of multicellular red algae, Florideophyceae, which includes *Eucheuma*, *Kappaphycus*, *Gracilaria*, and *Gelidium*, serve as economically important resources for carrageenan and agar (Lim et al., 2017). Volatile phytohormones, such as ethylene and methyl jasmonate, were reported to promote the formation of reproductive structures, cystocarp, and tetrasporangia as well as the release of their spores. These findings were already reviewed by Garcia-Jimenez and Robaina (2019), so it was not mentioned here.

The second group of multicellular red algae, Bangiophyceae, contains *Neopyropia*, *Porphyra*, and *Pyropia*. These are some of the most important marine aquaculture crops commonly used to wrap sushi and onigiri. The life cycle of Bangiophyceae generally consists of a heterogeneous alternation of macroscopic leaf-like gametophytes and microscopic filamentous sporophytes. During the sexual life cycle of Bangiales, blade gametophytes bear non-flagellated male (spermatia) and female (carpogonia) gametes. Fertilization occurs when female gametes are retained on the gametophyte, and successive cell divisions produce carpospores that then grow into filamentous sporophytes that are referred to as conchocelis; this was regarded as a different species before the clarification of its life cycle (Drew, 1949; Kurogi, 1953; Iwasaki, 1961). Conchocelis, which has a shell-borne lifestyle, appears to mitigate in grazing pressure during summer, which is the time of heavy herbivory.

Recent research showed that the application of 1-aminocylopropane-1-carboxylic acid (ACC), an ethylene precursor, induces the gametophytes to form spermatangia at the apical part and slightly lose photosynthetic pigments in monoecious species *Neopyropia yezoensis* (Uji et al., 2016), which is the main species cultivated for nori sheet in Japan. ACC also promotes the formation of spermatangia and parthenosporangia in male and female gametophytes, respectively, in dioecious species *Pyropia pseudolinearis* (Yanagisawa et al., 2019), which is harvested as food in a local area in Japan. ACC has also been widely used to replace ethylene treatment because the exogenous application of ACC can greatly increase ethylene production in higher plants (Elías et al., 2018; Sun et al., 2019). Likewise, the release of ethylene was increased by the addition of ACC in *N. yezoensis* (Uji et al., 2016), and it was expected that ethylene might be involved in *Pyropia/Neopyropia* reproduction. However, later research showed that the exogenous application of α-aminoisobutyric acid (AIB), a structural analog of ACC that blocks the conversion of ACC to ethylene in higher plants, mimics the effect of ACC to induce sexual reproduction without endogenous ACC accumulation (Endo et al., 2021). Meanwhile, 1-aminocyclobutane-1-carboxylic acid (ACBC), an exogenous ACC analog, promotes sexual reproduction in the same manner as ACC, whereas ethephon, an ethylene-releasing agent, does not (Uji et al., 2020). Therefore, these results raise an intriguing possibility that ACC plays a role as a signaling molecule independent from its role in ethylene signaling for sexual reproduction regulation in *Pyropia/Neopyropia*.

Although the signal transduction pathways of plant hormones remain obscure in macroalgae, a recent study provides evidence that phospholipase D (PLD) and phosphatidic acid (PA) are required for signal transduction events leading to ACC-induced sexual reproduction in *N. yezoensis* (Uji et al., 2022). PLD hydrolyzes membrane lipids, producing PA, which is now regarded as a lipid signaling molecule that regulates numerous physiological processes in eukaryotes (Wang, 2005; Li et al., 2009). In *N. yezoensis*, PLD activity and PA amount increase in response to ACC. Furthermore, the pharmacological inhibition of PLD by 1-butanol, an antagonist of PLD-dependent PA production, blocks ACC-induced spermatangia and carpospore production and prevents ACC-induced growth inhibition (Uji et al., 2022). Consistent with this observation, 1-butanol treatment inhibits the transcript accumulation of genes upregulated by ACC, including extracellular matrix-related genes, and alleviates the transcriptional decrease in genes downregulated by ACC, including photosynthesis-related genes (Uji et al., 2022). In higher plants, there are accumulating reports that PLD and its product PA mediate the signaling of various plant hormones, including ABA, ethylene, jasmonic acid, and salicylic acid (Munnik, 2001; Zhao, 2015). Likewise, PLD-PA signaling for ACC signaling may play an important role in the sexual reproduction of Bangiophyceae.

Previous studies showed that the exogenous application of ACC and ACC analogs enhances the tolerance of *N. yezoensis* gametophytes to hydrogen peroxide (H_2_O_2_) and heat stress (Uji et al., 2016; Uji et al., 2020; Uji and Mizuta, 2022). In contrast, ABA and salicylic acid application fails to mitigate the negative effects of heat stress on gametophytes (Uji and Mizuta, 2022). In photosynthetic organisms, the control of reactive oxygen species (ROS), such as superoxide and H_2_O_2_ accumulation in chloroplasts, is very important for their survival because the ROS produced in chloroplasts can cause irreversible oxidative damage, leading to serious damage to photosynthetic apparatus, particularly in photosystem II (PSII) (Suzuki et al., 2012; Gururani et al., 2015; Wang et al., 2018). Previous studies revealed that *N. yezoensis* gametophytes treated with ACC mitigate the decrease in *F*v/*F*m, the maximum quantum efficiency of photosystem II (PSII) photochemistry, under oxidative and heat stress (Uji et al., 2016; Uji et al., 2020; Uji and Mizuta, 2022). In addition, *NyHLIP* transcripts encoding a homolog of high-light-inducible protein (HLIP) of cyanobacteria as well as ascorbate (AsA), a non-enzymatic antioxidant, are increased during sexual reproduction induced by ACC treatment (Uji et al., 2020; Uji and Mizuta, 2022). The HLIP of cyanobacteria plays a protective role against singlet oxygen generation to prevent PSII photoinactivation (Sinha et al., 2012; Komenda and Sobotka, 2016). AsA, which is abundant in chloroplasts, is known to function as an electron donor for PSII, so it can protect PSII from inactivation under high temperature and high light conditions in higher plants (Tóth et al., 2011). *N. yezoensis* gametophytes generally produce spermatia and carpogonia at the beginning of spring. After fertilization, liberated carpospores germinate into sporophytes that grow during summer after being exposed to high light and heat stress. These findings suggest that an increase in HLIP and AsA during sexual reproduction may protect against PSII photoinactivation, which is critical to the acclimation of sporophytes to the habitat.

## Link between ROS and reproduction

Although ROS can cause oxidative damage to cells during abiotic and biotic stresses, ROS were found to regulate the development, cell proliferation, redox levels, stress, and plant hormone signaling in plants (Schmidt and Schippers, 2015; Mittler, 2017). For example, previous studies showed that decreasing the ROS levels of plant cells to below a particular threshold could result in a suppressed cellular proliferation (Mittler, 2017). In macroalgae, a high intracellular ROS production was observed during zoosporangium formation and paraphyses elongation in *S. japonica* (Mizuta and Yasui, 2010). Its sorus had significantly higher activities of ROS scavenging enzymes, such as ascorbate peroxidase, catalase (CAT), superoxide dismutase, and glutathione reductase compared to those of adjacent non-sorus blade sectors (Mizuta and Yasui, 2010). The high activity of ROS scavenging enzymes in the sorus may contribute to control the ROS levels and ensure the proper progression of reproduction.

Similarly, ROS generation in *N. yezoensis* gametophytes treated with ACC was observed to be accompanied by an increase in transcripts of respiratory burst oxidase homolog (Rboh) gene (Uji et al., 2020) that encodes nicotinamide adenine dinucleotide phosphate (NADPH) oxidases, which are the key producers of ROS. Packets of 128 spermatia are generally produced from a vegetative cell of *N. yezoensis* gametophytes by a series of mitotic cell divisions, while 16 carpospores are formed in a carpogonium after fertilization. Thus, a high amount of ROS during sexual reproduction may play an important role in cell proliferation to ensure the production of gametes and spores, in a particular spermatia. ROS also may contribute to the loosening of cell walls during reproduction to release gametes and spores because ROS plays a role in cleaving cell wall polysaccharides, causing the wall to loosen (Passardi et al., 2004). Among ROS scavenging enzymes, the mRNA levels of the CAT gene, which encodes catalase, are increased in mature gametophytes induced by ACC treatment (Uji et al., 2016).

In addition to ROS, previous studies presented evidence of the multifunctional role of AsA, such as in the regulation of cell division, cell expansion, and cell wall modification (Smirnoff, 1996). AsA is a crucial molecular modulator involved in cell progressing to S phase from G1 in root meristems and pericycle (Liso et al., 1988; Arrigoni et al., 1989). AsA can also act to the generated OH to cleave polysaccharides, leading to cell wall loosening (Fry, 1998). The increase in AsA during sexual reproduction in *N. yezoensis* appears to be a key player in forming gametes and spores and in cleaving cell wall polysaccharides for the liberation of gametes and spores, in addition to its role as a protection against abiotic stresses.

**Figure 1:**
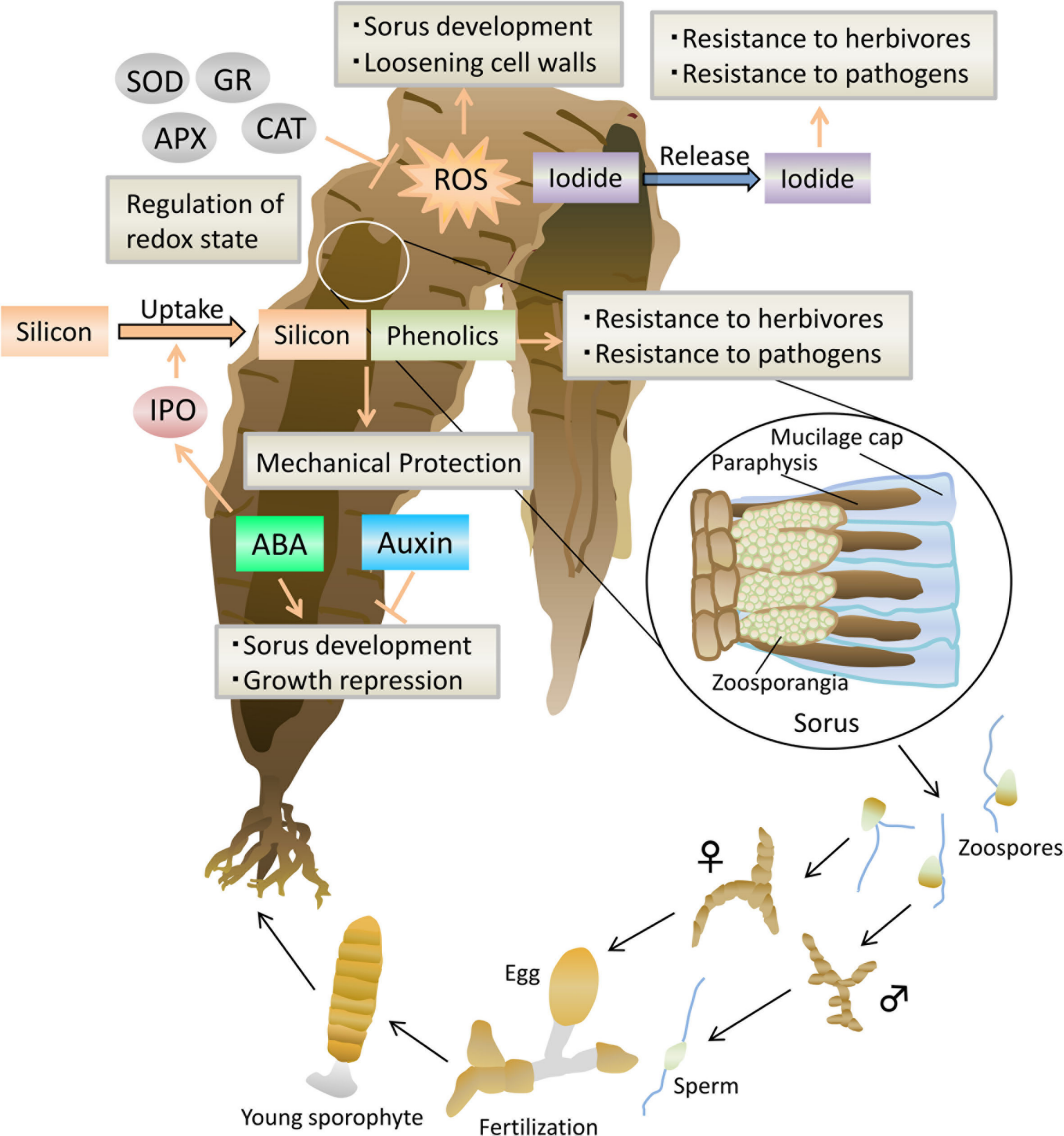
Model of the defense system during sorus development in *Saccharina japonica.* Arrows represent positive regulation, and blocked arrows represent negative regulation. ABA, abscisic acid; ROS, reactive oxygen species; IPO, iodoperoxidase; APX, ascorbate peroxidase; CAT, catalase; SOD, superoxide dismutase; GR, glutathione reductase.

**Figure 2:**
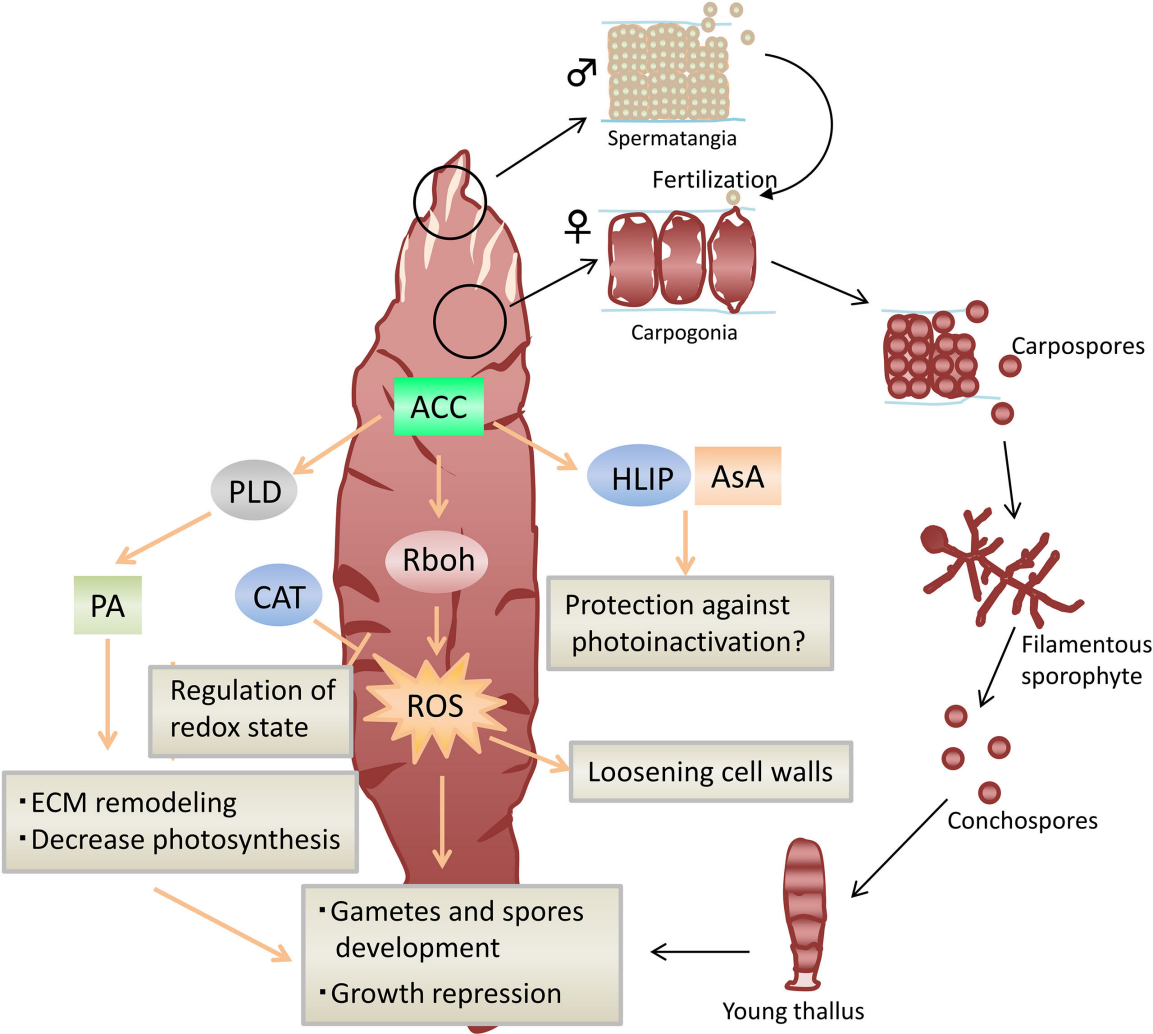
Model of ACC-mediated stress tolerance and sexual reproduction in *Neopyropia yezoensis.* Arrows represent positive regulation, and blocked arrows represent negative regulation. ACC, 1-aminocylopropane-1-carboxylic acid; ROS, reactive oxygen species; CAT, catalase; PLD, phospholipase D; PA, phosphatidic acid; Rboh, respiratory burst oxidase homolog; HLIP, high-light-inducible protein; AsA, ascorbate.

Based on the above, we propose a possible model of the regulatory mechanisms on the transition from vegetative to reproductive phase in Laminariales (**Figure 1**) and Bangiales (**Figure 2**).

## Conclusion and perspectives

Recent studies suggest that ABA and ACC trigger the switch from vegetative to reproductive growth in Laminariales and Bangiales, respectively. So far, the knowledge on the roles of ABA has been accumulated in land plants (Brookbank et al., 2021). However, ABA is present and functions in various organisms, including algae, suggesting that ABA has a functional significance in diverse organisms (Takezawa et al., 2011). ACC also appears to be a universal and ancient plant growth regulator according to recent research (Van de Poel, 2020), although its roles remain insufficiently understood. For a long time, ACC has only been noted for its function as an ethylene precursor. However, in recent years, a growing body of evidence indicates its role as a signaling molecule distinct from its role in ethylene biosynthesis in land plants (Polko and Kieber, 2019). In addition, the findings on the effects of ACC on red algae suggest that ACC signaling independent from ethylene is an ancient pathway conserved after years of evolution (Van de Poel, 2020). Thus, elucidating the functions and signaling pathways of ABA and ACC in various macroalgae is needed to clarify the mechanisms that regulate the switch between the vegetative and reproductive phases and the evolutionary perspective of plant hormones.

Further investigations are also needed to elucidate how environmental factors that influence their reproduction are involved in intrinsic factors, such as plant hormones ABA and ACC. Temperature and photoperiod are major important factors that influence the timing of seasonal reproduction in macroalgae (Brawley and Johnson, 1992; Agrawal, 2012; Liu et al., 2017). Hence, a systematic literature search implies that warming caused by anthropogenic climate change may result in a mismatch between temperature and light requirements, possibly leading to negative effects on their reproduction (De Bettignies et al., 2018). To date, there is an accumulating knowledge on mechanisms how macroalgae adapt to changes in temperature, particularly at high temperature (Heinrich et al., 2012; Sun et al., 2015; Wang et al., 2018; Uji et al., 2019; Zheng et al., 2020). In contrast, the photoperiod measuring system, which is a mechanism how macroalgae can count day or night lengths, is still unclear. A recent study reported that histone modification proteins were identified as potential key players that regulate diurnal rhythmic genes (Kominami et al., 2022). Thus, the elucidation of chromatin regulatory mechanisms related to photoperiod and plant hormone pathways may help unravel the mechanisms underlying the seasonal control of their reproduction. In addition to temperature and photoperiod, nutrients also influence macroalgal reproduction. For example, the seasonality of sorus formation in *S. japonica* was linked closely to the nitrogen content of sporophytes (Mizuta et al., 1998). In laboratory culture experiments, sorus formation in *S. japonica* was delayed in a nutrient-poor medium (Mizuta et al., 1999), and the sorus area increased with the accumulation of nitrogen or phosphorus (Nimura et al., 2002). The progress of research on the coordination between nutritional and hormonal signaling may be important in understanding the molecular mechanisms of macroalgal reproduction.

In Laminariales and Bangiales, growth and reproduction showed a negative correlation, while reproduction and stress tolerance showed a positive correlation, indicating that the success of their reproduction is required to allocate limited resources to them adequately. It is important to explore the cross-talk between ROS and plant hormones to help understand the mechanisms how macroalgae can invest their energy in growth, reproduction, and defense against biotic and abiotic stresses. A better understanding of mechanisms of trade-off may provide opportunities to design new breeding strategies that favor algal productivity and quality, such as by developing high-yielding cultivars by manipulating the resource allocation of reproduction and defense activation to growth.

## Author contributions

TU and HM wrote the manuscript. All authors contributed to the article and approved the submitted version.

## Funding

This study was supported by a grant-in-aid for Scientific Research (KAKENHI) (20K06176 to HM, 22K05779 to TU) from the Japan Society for the Promotion of Science.

## Conflict of interest

The authors declare that the research was conducted in the absence of any commercial or financial relationships that could be construed as a potential conflict of interest.

## Publisher’s note

All claims expressed in this article are solely those of the authors and do not necessarily represent those of their affiliated organizations, or those of the publisher, the editors and the reviewers. Any product that may be evaluated in this article, or claim that may be made by its manufacturer, is not guaranteed or endorsed by the publisher.
